# Nahrungsergänzungsmittel und angereicherte Lebensmittel: Nutzen, Risiken und Maßnahmen für den Verbraucherschutz

**DOI:** 10.1007/s00103-025-04134-1

**Published:** 2025-10-10

**Authors:** Carolin Bendadani, Nadiya Bakhiya, Evelyn Breitweg-Lehmann, Anke Ehlers, Karen Ildico Hirsch-Ernst, Birgit Liebscher, Anke Weißenborn

**Affiliations:** 1 Abteilung Lebensmittelsicherheit, Bundesamt für Verbraucherschutz und Lebensmittelsicherheit, Berlin, Deutschland; 2 Abteilung Lebens- und Futtermittelsicherheit in der Nahrungskette, Bundesinstitut für Risikobewertung, Max-Dohrn-Str. 8–10, 10589 Berlin, Deutschland

**Keywords:** Vitamine, Mineralstoffe, Mikronährstoffe, Risikobewertung, Lebensmittelsicherheit, Vitamins, Minerals, Micronutrients, Risk assessment, Food safety

## Abstract

In Deutschland nehmen etwa 2 Drittel der Erwachsenen und etwa 5–20 % der Kinder Nahrungsergänzungsmittel (NEM), oft in der Annahme, dadurch ihre Gesundheit oder Leistungsfähigkeit zu verbessern. Verzehrdaten zeigen, dass die Bevölkerung hierzulande mit wenigen Ausnahmen adäquate Mengen an Mikronährstoffen über die übliche Nahrung aufnimmt. NEM sind daher für gesunde Menschen, die sich abwechslungsreich und ausgewogen ernähren, in der Regel unnötig. Es gibt nur wenige Fälle, in denen eine gezielte Nahrungsergänzung sinnvoll sein kann. Dagegen erhöht die regelmäßige Nutzung von hochdosierten NEM, vor allem bei gut versorgten Menschen, das Risiko für unerwünschte Effekte, insbesondere wenn die Produkte auch „sonstige Stoffe“ mit ernährungsspezifischer oder physiologischer Wirkung enthalten wie etwa Omega-3-Fettsäuren oder Koffein.

Mikronähr- oder „sonstige Stoffe“ dürfen auch herkömmlichen Lebensmitteln zugesetzt werden. Die so „angereicherten Lebensmittel“ können – oft unbemerkt – erheblich zur Gesamtexposition beitragen. In der Europäischen Union (EU) sind NEM (wie auch angereicherte Lebensmittel) rechtlich als Lebensmittel, *nicht *als Arzneimittel definiert. Im Lebensmittelrecht gilt, dass Lebensmittel, die nicht sicher sind, nicht in Verkehr gebracht werden dürfen. Für die Sicherheit sind die Lebensmittelunternehmer verantwortlich; die Kontrolle obliegt in Deutschland den Landesüberwachungsbehörden. Dies stellt eine große Herausforderung dar, u. a., weil NEM zunehmend über das Internet vertrieben werden. Um die Überwachung zu erleichtern und den Schutz der Verbraucher zu gewährleisten, müssen dringend Regelungslücken geschlossen werden, indem EU-harmonisierte Höchstmengen für Mikronährstoffe festgesetzt und „sonstige Stoffe“ besser reguliert werden.

## Abstract (en)

In Germany, around two thirds of adults and 5–20% of children take food supplements, many hoping to improve their health or performance. Consumption data show that with a few exceptions the intake of micronutrients through conventional foods is adequate in Germany. Food supplements are thus generally not considered necessary for healthy people who follow a varied and balanced diet. There are thus only a few situations in which a food supplement can be useful. Routine use of high-dose supplements, above all by people with adequate nutrient supply, increases the risk of harm to health, especially if products also contain ‘other substances’ with a nutritional or physiological effect, such as omega‑3 fatty acids or caffeine.

Micronutrients or ‘other substances’ may also be added to conventional foods. Discretionarily fortified foods can—often without being noticed—contribute significantly to the overall exposure. In the European Union (EU), food supplements (as well as fortified foods) are legally defined as food, *not* as medicine. According to the Food Law, food shall not be placed on the market if it is unsafe. It is the responsibility of the food business operator to safeguard this, while in Germany the federal states (Länder) are responsible for food control and inspection. This is increasingly challenging due to the fact that a significant proportion of food supplements is marketed via the internet. In order to facilitate monitoring and ensure consumer protection, existing regulatory gaps must be closed, for example by setting EU-harmonised maximum amounts for vitamins and minerals and by improving the regulation of ‘other substances’.

## Einleitung

Der Konsum von Nahrungsergänzungsmitteln (NEM) ist in Deutschland weitverbreitet. Im Jahr 2023 setzten Apotheken rund 3,11 Mrd. € damit um. Die Produkte können Vitamine und/oder Mineralstoffe, aber auch „sonstige Stoffe“ mit ernährungsspezifischer oder physiologischer Wirkung (engl. „other substances“) enthalten, wie z. B. Omega-3-Fettsäuren, Aminosäuren oder Extrakte aus Pflanzen (sogenannte Botanicals). Bei Letzteren handelt es sich um eine Vielzahl von Stoffen, die oft nicht gut charakterisiert und in der Europäischen Union (EU) weitgehend unreguliert sind.

Etwa 60–75 % der Erwachsenen greifen zu NEM – davon etwa 30 % täglich oder mehrmals pro Woche [1–3]. Auch nehmen etwa 5 % der unter 6‑Jährigen [4] und 14–19 % der 12- bis 17-Jährigen regelmäßig NEM [5].

Bekannt ist, dass Frauen und Menschen mit höherem Bildungsstatus, gesünderem Lebensstil und ausgewogener Ernährung häufiger NEM einnehmen [6, 7]. Als Hauptgründe werden Gesundheitsvorsorge oder -verbesserung sowie der Wunsch nach einer Steigerung des Wohlbefindens, einschließlich Lebensqualität und Leistungsfähigkeit, genannt [5].

Mikronährstoffe und „sonstige Stoffe“ werden aber auch herkömmlichen Lebensmitteln zugesetzt. Die so angereicherten Lebensmittel unterscheiden sich meist weder im Aussehen noch im Geschmack von den nicht angereicherten Pendants, können aber zu einer höheren Nährstoffzufuhr beitragen [8, 9].

Vor diesem Hintergrund besteht eine große Herausforderung darin, einerseits NEM mit Mikronährstoffen und angereicherte Lebensmittel für Personen mit unzureichender Nährstoffzufuhr oder erhöhtem Bedarf an bestimmten Nährstoffen bereitzustellen und zugleich gut versorgte Bevölkerungsgruppen vor einer zu hohen Nährstoffzufuhr zu schützen. Dabei ist zu berücksichtigen, wie NEM definiert und rechtlich einzuordnen sind, welche Mengen an Mikronährstoffen über die übliche Ernährung aufgenommen werden, wie die Bevölkerung mit Nährstoffen versorgt ist und welche gesundheitlichen Effekte eine zu hohe oder zu geringe Zufuhr mit sich bringen kann, kurz: welche Risiken mit NEM und angereicherten Lebensmitteln verbunden sind und welche Maßnahmen geeignet sind, diese zu minimieren, um Verbraucher bestmöglich zu schützen.

## Lebensmittelrechtliche Bestimmungen

**Abb. 1:**
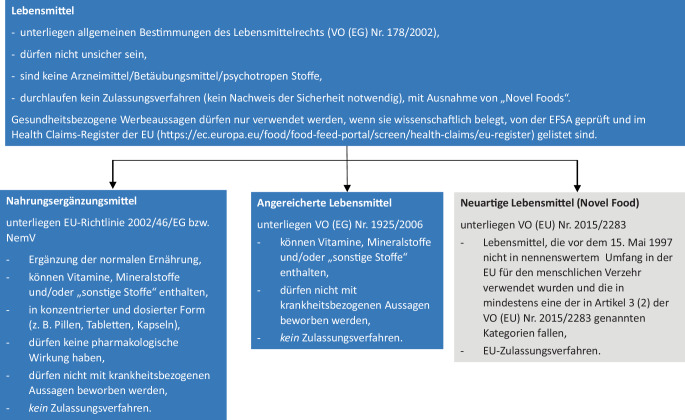
Definitionen und regulatorische Merkmale der verschiedenen Lebensmittelkategorien

Nahrungsergänzungsmittel und angereicherte Lebensmittel sind in der EU als Lebensmittel definiert, *nicht* als Arzneimittel (siehe auch Beitrag von Schulze in diesem Themenheft). Gemäß Basisverordnung VO (EG) Nr. 178/2002 [10] sind beim Herstellen und Inverkehrbringen von Lebensmitteln bestimmte Anforderungen zu erfüllen (Abb. 1).

Mit Ausnahme von „neuartigen Lebensmitteln“ werden Lebensmittel, einschließlich NEM, vor dem Inverkehrbringen *nicht* behördlich geprüft oder zugelassen. Primär sind die Lebensmittelunternehmer für die Sicherheit verantwortlich. Die Überwachung, einschließlich Kontrolle der Kennzeichnung und Einhaltung lebensmittelrechtlicher Bestimmungen, erfolgt in Deutschland durch die Lebensmittelüberwachung der Bundesländer.

### Nahrungsergänzungsmittel.

Gemäß Richtlinie 2002/46/EG [11], die mit der Verordnung über Nahrungsergänzungsmittel (NemV; [12]) in nationales Recht umgesetzt wurde, ist ein NEM ein Lebensmittel, das

1. dazu bestimmt ist, die *allgemeine Ernährung zu ergänzen*,
2. ein *Konzentrat von Nährstoffen* oder *sonstigen Stoffen mit ernährungsspezifischer oder physiologischer Wirkung* allein oder in Zusammensetzung darstellt und
3. *in dosierter Form*, insbesondere in Form von Kapseln, Pastillen, Tabletten, Pillen und anderen ähnlichen Darreichungsformen …, zur *Aufnahme in abgemessenen kleinen Mengen* in den Verkehr gebracht wird.

### Angereicherte Lebensmittel.

Nach VO (EG) Nr. 1925/2006 (Anreicherungs-Verordnung) dürfen Vitamine und/oder Mineralstoffe oder „sonstige Stoffe“ auch Lebensmitteln des allgemeinen Verzehrs zugesetzt werden, unabhängig davon, ob sie normalerweise darin enthalten sind oder nicht. Die Stoffe sollen in einer für den Menschen bioverfügbaren Form zugesetzt werden. Ein Zusatz kann erfolgen, wenn

- in der Bevölkerung … ein Mangel an … Vitaminen und/oder Mineralstoffen besteht, …,
- die Möglichkeit besteht, den Ernährungszustand der Bevölkerung … zu verbessern und/oder … mögliche Defizite … zu beheben, oder
- sich die allgemein anerkannten wissenschaftlichen Kenntnisse über die Bedeutung von Vitaminen und Mineralstoffen in der Ernährung und deren gesundheitliche Auswirkungen weiterentwickelt haben [13].

### Mikronährstoffe und sonstige Stoffe.

Nur die im Anhang I der Richtlinie 2002/46/EG [11] bzw. VO (EG) Nr. 1925/2006 [13] aufgeführten Vitamine und/oder Mineralstoffe dürfen in den in Anhang II gelisteten Formen verwendet werden.

Wird NEM oder anderen Lebensmitteln ein „sonstiger Stoff“ zugesetzt und sind dadurch Zufuhrmengen zu erwarten, die weit über den bei ausgewogener und abwechslungsreicher Ernährung zu erwartenden liegen und/oder die ein potenzielles gesundheitliches Risiko bergen, kann der betreffende Stoff gemäß Artikel 8 Absatz 5 der VO (EG) Nr. 1925/2006 in den Anhang III dieser VO aufgenommen werden – und zwar entweder in

- Teil A (Stoffe, die gesundheitsschädlich und damit verboten sind) oder
- Teil B (Stoffe, deren Zusatz/Verwendung nur unter den genannten Bedingungen erlaubt ist).

Sofern der Stoff *möglicherweise *gesundheitsschädlich ist, jedoch eine wissenschaftliche Unsicherheit besteht, kann er in

- Teil C (Stoffe, die von der Gemeinschaft geprüft werden, da die aktuelle Datenlage für eine Einstufung nicht ausreicht)

aufgenommen werden [13].

Im Falle der Einordnung in Teil C ist binnen 4 Jahren zu entscheiden, ob die Verwendung des Stoffes allgemein erlaubt wird oder eine Zuordnung in Teil A oder B erfolgen soll [13]. Ein aktuelles Beispiel für eine innerhalb der 4‑Jahres-Frist durchgeführte wissenschaftliche Bewertung, bei der die geäußerten Sicherheitsbedenken von der Europäischen Behörde für Lebensmittelsicherheit (EFSA) bestätigt wurden, sind Monacoline aus Rotschimmelreis (englisch: „red yeast rice“ (RYR)). Auf Basis der vorgelegten Daten war es der EFSA nicht möglich, die Sicherheit von Monacolinen aus Rotschimmelreis bei Dosierungen unter 3 mg pro Tag in NEM festzustellen oder eine tägliche Zufuhr zu ermitteln, bei der keine Sicherheitsbedenken bestehen [14]. Folglich ist zu erwarten, dass die EU-Kommission (KOM) Monacoline aus Rotschimmelreis in Teil A des Anhangs III der VO (EG) Nr. 1925/2006 aufnimmt und diese somit verboten werden.

Darüber hinaus wird auf EU-Ebene zurzeit über die Verwendung von Pflanzen/-teilen, die Hydroxyanthrancenderivate (HAD) enthalten, diskutiert. So wurden beim Europäischen Gerichtshof (EuGH) 4 Klagen gegen die Aufnahme bestimmter HAD und Zubereitungen, die HAD enthalten, in Teil A und C des Anhangs III der VO (EG) Nr. 1925/2006 eingereicht [15–18]. Die EuGH-Urteile sind noch nicht rechtskräftig; sie hätten aber zur Folge, dass alle Einträge zu HAD, außer Danthron, im Anhang III der Verordnung aufgehoben würden. Gegen diese teilweise Nichtigerklärung wurde von der KOM Revision eingelegt und klargestellt, dass die Vorgaben der VO (EG) Nr. 1925/2006 bezüglich HAD solange Anwendung finden, wie keine finale Entscheidung des EuGH vorliegt [19–22].

Ungeachtet dessen enthält der Anhang III bis heute (Stand: 09/2025) nur sehr wenige Einträge, was dazu führt, dass es für die Verwendung „sonstiger Stoffe“ in der EU zurzeit keine harmonisierten und insgesamt kaum Regelungen gibt. Für alle nicht im Anhang III gelisteten „sonstigen Stoffe“ sind weiterhin nationale Vorschriften anzuwenden und Einzelfallprüfungen auf Basis der allgemeinen Regelungen des Lebensmittelrechts durchzuführen. In vielen Fällen ist jedoch die wissenschaftliche Datenlage nicht ausreichend, um Aussagen darüber treffen zu können, ob ein Stoff ein Gefährdungspotenzial besitzt oder nicht.

## Nahrungsergänzungsmittel – wann sind sie sinnvoll?

Per definitionem sind NEM dazu bestimmt, die *allgemeine Ernährung* zu ergänzen. Dies kann unter bestimmten Bedingungen bzw. bei einzelnen Mikronährstoffen angezeigt sein – z. B. wenn die Zufuhr durch die übliche Nahrung nicht ausreicht. Unter „ausreichender“ Zufuhr wird hier verstanden, dass die Zufuhrreferenzwerte der Deutschen Gesellschaft für Ernährung (DGE) erreicht werden. Die DGE berücksichtigt bei der Referenzwertableitung endogene und exogene Faktoren sowie neue wissenschaftliche Erkenntnisse. Unter einer „ausreichenden“ Zufuhr ist also nicht allein die Bedarfsdeckung zur Vermeidung von Mangelerscheinungen zu verstehen, sondern eine Zufuhrmenge, die auch gesundheitsförderliche Aspekte einzelner Mikronährstoffe berücksichtigt.

Zudem können bestimmte NEM aus präventivmedizinischer Sicht oder für bestimmte Bevölkerungsgruppen wie Schwangere und/oder Stillende empfohlen werden (Tab. 1). Allerdings gibt es hier und auch allgemein keine wissenschaftlichen Belege dafür, dass eine über den Bedarf hinausgehende Zufuhr von Mikronährstoffen einen Nutzen für die Gesundheit hat [23, 24].

**Tab. 1:** Empfehlungen zur Mikronährstoffsupplementierung in besonderen Lebensphasen

Säuglinge [40]	Jeder Säugling soll zusätzlich zu Muttermilch oder Säuglingsanfangs- oder Folgenahrung Vitamin K, Vitamin D und Fluorid erhalten	Es sollen 3‑mal 2 mg Vitamin K als Tropfen bei den Vorsorgeuntersuchungen U1, U2 und U3 gegeben werden. Alternativ kann das Vitamin in besonderen Situationen einmalig durch eine Vitamin-K-Injektion ärztlich verabreicht werden
		Es sollen täglich 400–500 IE (10–12,5 μg) Vitamin D als Tablette oder Tropfen bis zum erlebten 2. Frühsommer, d. h. je nach Geburtszeitpunkt für etwa 12 bis 18 Monate, gegeben werden, bis eine stärkere Vitamin-D-Eigensynthese bei Sonnenlichtexposition erfolgt
		Kombiniert mit der Vitamin-D-Gabe sollen bis zum Zahndurchbruch täglich 0,25 mg Fluorid zur Kariesprophylaxe gegeben werden
Frauen vor und in der Schwangerschaft und Stillzeit [38]	Eine adäquate Jodzufuhr soll bei Frauen in Schwangerschaft und Stillzeit sichergestellt werden	Milch und Milchprodukte sowie Meeresfisch^a^ sollten regelmäßig verzehrt werden
		Es sollte im Haushalt ausschließlich jodiertes Speisesalz verwendet werden
		Mit jodiertem Speisesalz hergestellte Lebensmittel sollten bevorzugt werden
		Es sollten täglich 100 µg (bis 150 µg) Jod supplementiert werden (nach vorheriger Jodanamnese)
	Frauen, die schwanger werden wollen oder könnten, sollten zusätzlich zu einer folatreichen Ernährung Folsäure supplementieren, um das Risiko eines Neuralrohrdefekts beim Kind zu reduzieren	Neben einer folatreichen Ernährung sollten Frauen vor und in der Schwangerschaft täglich 400 µg Folsäure (oder eine äquivalente Menge anderer Folate) supplementieren. Die Supplementierung sollte mindestens 4 Wochen vor der Konzeption beginnen und bis zum Ende der 12. Schwangerschaftswoche fortgesetzt werden

^a^ Ein hoher Verzehr an Raubfischarten (zum Beispiel Thunfisch, Schwertfisch), die am Ende der maritimen Nahrungskette stehen und höhere Gehalte an gesundheitlich bedenklichen Stoffen aufweisen können, sollte von Schwangeren vermieden werden

**Abb. 2:**
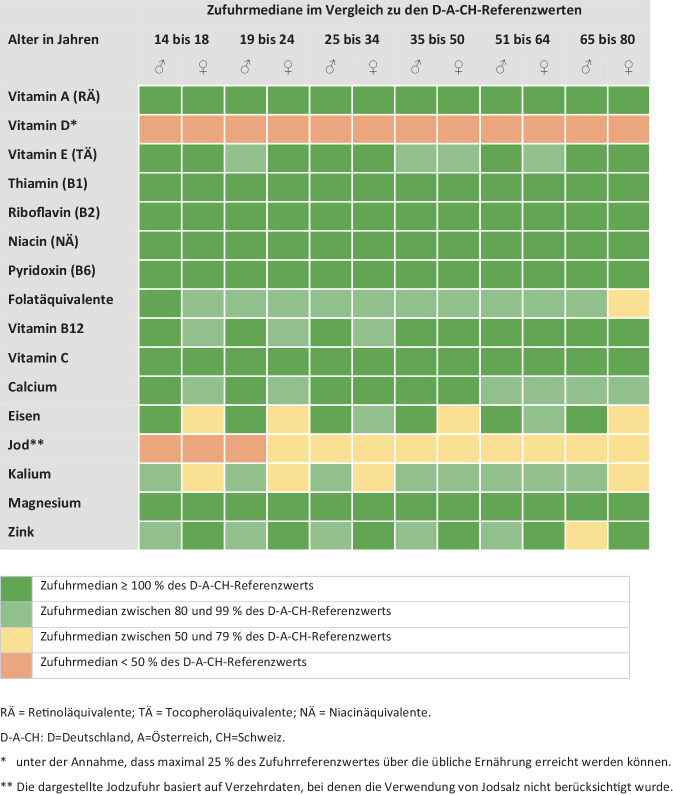
Risiko einer Unterversorgung mit Vitaminen und Mineralstoffen bei Jugendlichen und Erwachsenen in Deutschland, bei herkömmlicher Ernährung gemäß NVS II [25]

**Abb. 3:**
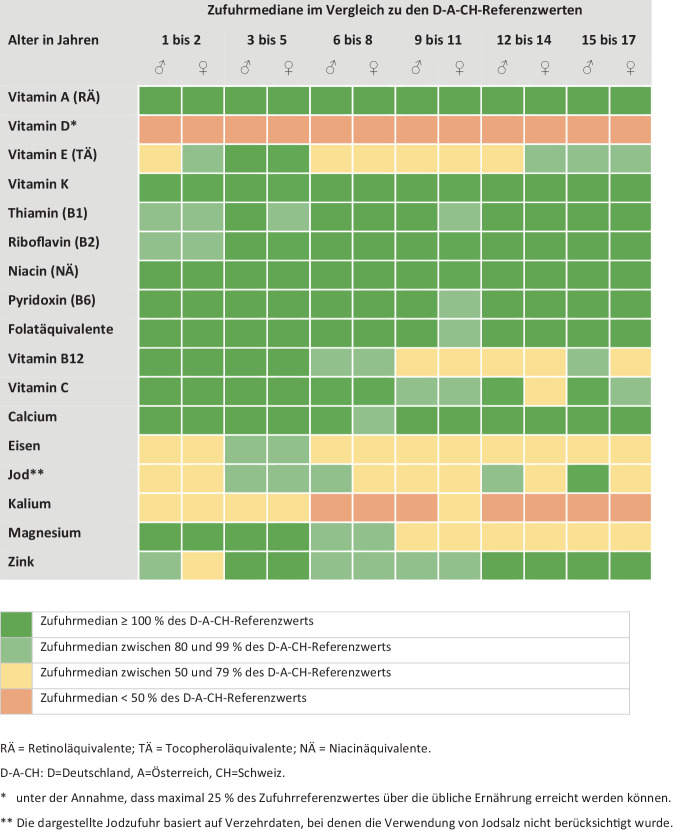
Risiko einer Unterversorgung mit Vitaminen und Mineralstoffen bei Kindern und Jugendlichen in Deutschland, bei herkömmlicher Ernährung gemäß KiESEL (1- bis 5‑Jährige; [26]) und EsKiMo II (6- bis 17-Jährige; [27])

Die für Deutschland vorliegenden Daten aus der Nationalen Verzehrstudie (NVS) II (Erwachsene) sowie aus KiESEL^1^ (Kinder von 0,5 bis 5 Jahren) und EsKiMo^2^II (Kinder von 6 bis 17 Jahren) belegen, dass die Zufuhr von Vitaminen und Mineralstoffen im Allgemeinen ausreichend ist [25–27]. Mikronährstoffe, bei denen die Zufuhrmediane der Erwachsenen unter den jeweiligen D‑A-CH-Referenzwerten (D = Deutschland, A = Österreich und CH = Schweiz) liegen, sind Vitamin D, Jod sowie teilweise Eisen (bei Frauen) und teilweise Kalium (Abb. 2; [25]). Bei Kindern und Jugendlichen sind es Vitamin D sowie teilweise Vitamin E, Vitamin B12, Eisen, Jod, Kalium und bei Kindern ab 9 Jahren Magnesium (Abb. 3; [26, 27]).

### Vitamin D.

Bei Vitamin D ist zu berücksichtigen, dass herkömmliche Lebensmittel nur relativ geringe Vitamin-D-Gehalte aufweisen und Zufuhrdaten daher nicht geeignet sind, den Versorgungsstatus verlässlich zu beurteilen. Statusdaten aus KiGGS^3^ und DEGS 1^4^ deuten darauf hin, dass etwa 54 % der Kinder und Jugendlichen sowie 44 % der Erwachsenen in Deutschland adäquate 25-OH-D-Serumspiegel ≥50 nmol/L (20 ng/ml) erreichen [28].

Allerdings zählen Bewohner von Pflegeeinrichtungen, die meist mobilitätseingeschränkt sind und sich daher wenig im Freien aufhalten, zu den Risikogruppen für einen unzureichenden Vitamin-D-Status. Bei ihnen kann eine generelle Vitamin-D-Gabe über Supplemente erwogen werden [29, 30]. Auch kann bei anderen Personen, die sich entweder kaum oder gar nicht im Freien aufhalten, die nur mit gänzlich bedecktem Körper nach draußen gehen oder dunkelhäutig sind, eine Vitamin-D-Supplementierung – nach vorheriger Statusbestimmung – angezeigt sein [31]. Wer vorbeugend Vitamin D supplementieren möchte, sollte auf NEM mit bis zu 20 µg Vitamin D (800 IE) zurückgreifen. Damit werden adäquate Serumspiegel erzielt [32] und selbst bei chronischer Einnahme keine adversen Effekte beobachtet [33].

### Jod.

Ein anderer Stoff, bei dem in Deutschland ein erhöhtes Risiko für eine unzureichende Versorgung besteht, ist Jod. Aktuelle repräsentative Daten deuten darauf hin, dass die Jodversorgung der deutschen Bevölkerung nicht optimal oder sogar rückläufig ist [34, 35]. Aus Sicht der Risikobewertung sollte dem durch eine Erhöhung des Verwendungsgrades von Jodsalz in verarbeiteten Lebensmitteln bei weiterer Nutzung von Jodsalz im Haushalt begegnet werden [36]. Ein direkter Zusatz von Jod zu Lebensmitteln oder Jodsupplemente werden für die Allgemeinbevölkerung nicht empfohlen. Nur in Schwangerschaft und Stillzeit wird – nach vorheriger Jodanamnese zur Vermeidung einer übermäßigen Zufuhr aus weiteren Quellen – eine Supplementierung von 100 µg (bis 150 µg) Jod pro Tag empfohlen [36–38].

### Vegane Ernährung.

Eine gezielte Nahrungsergänzung mit bestimmten Mikronährstoffen kann auch bei besonderen Ernährungsweisen sinnvoll sein, so zum Beispiel bei veganer Ernährung, bei der auf tierische Lebensmittel gänzlich verzichtet wird und daher ein hohes Risiko für eine Unterversorgung mit Mikronährstoffen besteht, die in pflanzlichen Lebensmitteln nur in geringen Mengen enthalten oder weniger gut bioverfügbar sind. Neben Vitamin B12 und Jod gelten bei einer veganen Ernährung die Vitamine D, B2 und ggf. Vitamin A sowie Calcium, Eisen, Zink und Selen, aber auch langkettige Omega-3-Fettsäuren als (potenziell) kritische Nährstoffe [39]. Insbesondere in Phasen von Wachstum und Entwicklung sind die Deckung des Proteinbedarfs und auch die Versorgung mit Eisen, Calcium, Jod und Vitamin B12 bei vollständigem Verzicht auf tierische Lebensmittel schwierig. Eine Supplementierung von Vitamin B12 ist daher bei veganer Ernährung zwingend erforderlich; weitere Mikronährstoffe sind bei Bedarf zu supplementieren. Insbesondere in der frühen Kindheit wird geraten, die Nährstoffversorgung sowie die altersgerechte Entwicklung regelmäßig ärztlich-diagnostisch überprüfen zu lassen [40].

## Gesundheitliche Risiken durch NEM

Nahrungsergänzungsmittel werden prinzipiell ohne Zulassungsverfahren auf den Markt gebracht. Auch gibt es kaum systematische Studien über unerwünschte Wirkungen. Erschwerend kommt hinzu, dass viele Produkte über das Internet gehandelt werden und sich international angesiedelte Onlineshops der Überwachung entziehen können. Zum Teil sind sich weder NEM-Konsumenten noch deren betreuende Ärzte über die Einnahme von NEM und gesundheitliche Risiken, die sich daraus ergeben können, bewusst. Publizierte Fallberichte deuten darauf hin, dass adverse Effekte u. a. aufgrund von Fehlanwendung und/oder unkritischer Einnahme von NEM auftreten können. Allerdings gibt es zurzeit weder in Deutschland noch in der EU ein verpflichtendes Meldesystem dafür.

### Risiken einer zu hohen Mikronährstoffzufuhr

**Abb. 4:**
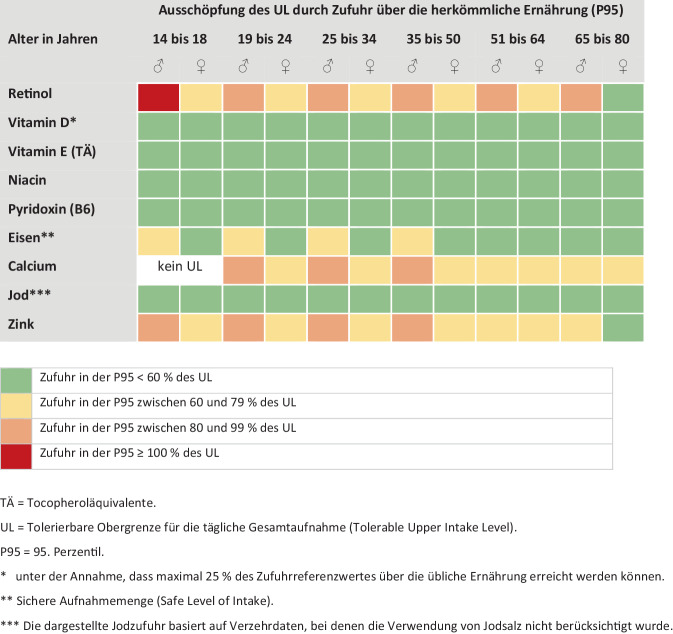
Risiko einer Überversorgung mit Vitaminen und Mineralstoffen bei Jugendlichen und Erwachsenen bei herkömmlicher Ernährung, gemäß NVS II [25]

Durch den Verzehr herkömmlicher Lebensmittel ist eine zu hohe Zufuhr von Mikronährstoffen und eine dadurch bedingte Überversorgung nahezu ausgeschlossen. Die vorliegenden Daten deuten jedoch darauf hin, dass bei einigen Nährstoffen bereits durch die normale Ernährung in hohen Verzehrperzentilen (95. Perzentil, P95) Zufuhrmengen erreicht werden, die mehr als 60 % der tolerierbaren Obergrenze für die tägliche Gesamtaufnahme (Tolerable Upper Intake Level, UL)^5^ ausschöpfen oder überschreiten (Abb. 4 und 5). Zwar führt eine Überschreitung des UL nicht zwangsläufig zu unerwünschten Effekten; sie erhöht aber das Risiko dafür [41]. Werden zusätzlich (hoch dosierte) NEM eingenommen und/oder angereicherte Lebensmittel verzehrt, steigt das Risiko für eine Überversorgung mit den jeweiligen Vitaminen und/oder Mineralstoffen. Ein Beispiel für unerwünschte Effekte durch zu hohe und unkontrollierte Supplementeinnahme ist *Vitamin D*.

**Abb. 5:**
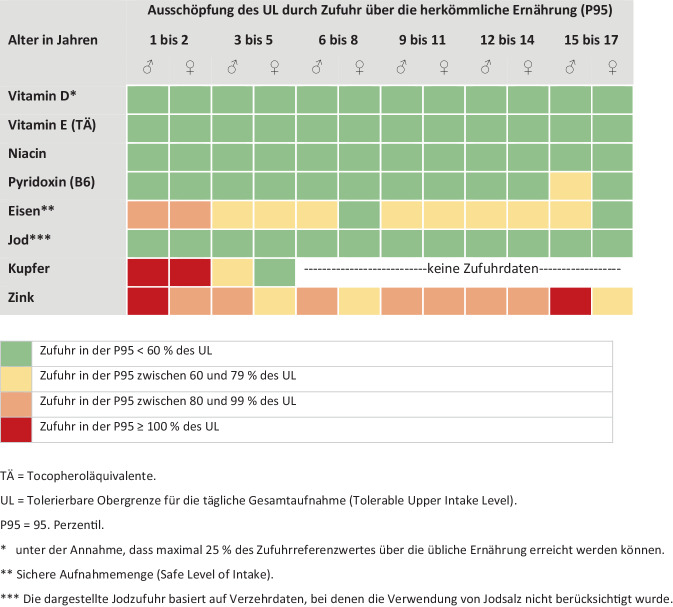
Risiko einer Überversorgung mit Vitaminen und Mineralstoffen bei Kindern und Jugendlichen bei herkömmlicher Ernährung, gemäß KiESEL (1- bis 5‑Jährige; [26]) und EsKiMo II (6- bis 17-Jährige; [27])

In den für Deutschland bekannten, d. h. publizierten Fällen von Vitamin-D-Intoxikationen wurde über einen längeren Zeitraum täglich Vitamin D in Dosierungen von etwa 10.000 IE (250 µg) bis 60.000 IE (1500 µg) eingenommen (IE=Internationale Einheiten). Die daraus folgenden schweren Vitamin-D-Intoxikationen gingen mit Nierenverkalkung, akutem Nierenversagen oder sogar einer irreversiblen dialysepflichtigen Niereninsuffizienz einher. Betroffen waren neben Erwachsenen [42–44] auch Säuglinge und Kleinkinder, denen von ihren Eltern in guter Absicht exzessive Mengen an Vitamin D verabreicht worden waren [45, 46]. Diese führten zu extrem hohen 25-Hydroxy-Vitamin-D-(25-OH-D-)-Spiegeln (z. T. > 1500 nmol/l; 600 ng/ml; [45]), weshalb die klassischen Symptome einer akuten Vitamin-D-Intoxikation, wie z. B. stark ausgeprägte Hyperkalzämie, bereits nach relativ kurzer Zeit auftraten.

### Risiken durch NEM, die „sonstige Stoffe“ enthalten

In einer aktuellen repräsentativen Bevölkerungsbefragung gaben aber mehr als 10 % der NEM-Nutzer an, dass bei ihnen im Zusammenhang mit der NEM-Einnahme unerwünschte Wirkungen aufgetreten seien. Neben gastrointestinalen Beschwerden oder Kopfschmerzen waren dies auch Wechselwirkungen mit Medikamenten. Selbst wenn sich daraus keine Kausalzusammenhänge ableiten lassen, können mit der Einnahme von NEM gesundheitliche Risiken verbunden sein – z. B. wenn die ggf. enthaltenen „sonstigen Stoffe“ Gesundheitsgefahren bergen und/oder wenn Interaktionen mit Arzneimitteln auftreten (z. B. [47–50]). Im Folgenden werden 2 aktuelle Beispiele für unerwünschte Effekte durch NEM mit „sonstigen Stoffen“ vorgestellt.

#### Omega-3-Fettsäuren.

In systematischen Übersichtsarbeiten und Metaanalysen randomisierter placebokontrollierter Interventionsstudien wurde bei Patienten mit kardiovaskulären Erkrankungen oder Risikofaktoren dafür, die mit Omega-3-Fettsäure-haltigen Präparaten behandelt wurden, dosisabhängig ein erhöhtes Risiko für Vorhofflimmern beobachtet. Das Risiko war bei der höchsten getesteten Dosis von 4 g pro Tag am größten [51–54], wurde aber auch bei etwa 2 g pro Tag beobachtet, wenngleich mit grenzwertiger Signifikanz [52, 54]. Basierend auf diesen Beobachtungen wurde von einigen pharmazeutischen Unternehmen (Zulassungsinhaber von Omega-3-Fettsäure-haltigen Arzneimitteln) in Abstimmung mit dem Bundesinstitut für Arzneimittel und Medizinprodukte (BfArM) ein sogenannter Rote-Hand-Brief veröffentlicht, in dem über das Risiko für Vorhofflimmern bei Verwendung Omega-3-Fettsäure-haltiger Arzneimittel informiert wird [55]. Da NEM mit Omega-3-Fettsäuren freiverkäuflich auf dem Markt sind, sollten insbesondere Menschen mit Herzerkrankungen oder entsprechenden Risikofaktoren derartige Präparate nur nach ärztlicher Rücksprache einnehmen [56]. Darüber hinaus gibt es Hinweise darauf, dass eine Supplementierung mit hohen Dosen von Omega-3-Fettsäuren die Thrombozytenaggregation hemmen und Blutungen bei Patienten, die gerinnungshemmende Medikamente einnehmen, fördern kann [57, 58].

#### Koffein.

Koffeinhaltige Pulver werden teilweise auch als NEM angeboten [59] und unter anderem als „Pre-Workout“-Produkte zur Leistungssteigerung beworben. Insbesondere in einzelnen Online-Shops werden auch reine oder hochkonzentrierte Koffein-Pulver nicht portioniert und z. T. in großen Mengen als NEM vermarktet. Bei diesen Produkten ist zwar in der Regel die empfohlene Verzehrmenge angegeben, aber anders als bei eindeutig dosierten Koffein-Tabletten ist diese vom Konsumenten selbst abzumessen oder -wiegen. Da die gesundheitlich unbedenkliche Dosis von 0,2 g (als Einzeldosis) bei hochkonzentriertem oder reinem Koffein einer nur sehr geringen Pulvermenge entspricht, lässt sich diese mit einer herkömmlichen Küchenwaage (Messgenauigkeit erst ab etwa 1 g) nicht abwiegen [59]. Auch mit den zum Teil beigefügten Dosierlöffeln lässt sich eine derart geringe Pulvermenge nicht exakt abmessen. Überdies wissen sicher viele Menschen, wie sie auf eine Tasse aufgebrühten Pulverkaffee reagieren; dass aber die gleiche Menge reines Koffein eine immens höhere Wirkstärke hat, ist ihnen vielleicht nicht bekannt. Versehentlich können so extrem hohe Koffeindosen eingenommen werden, was bereits zu Fällen von schweren akuten Koffein-Vergiftungen geführt hat [60, 61]. So verstarb trotz intensivmedizinischer Behandlung eine junge Frau, die ohne Kenntnis der Dosierungsempfehlung versehentlich 2 Teelöffel hochkonzentriertes Koffeinpulver (ca. 9 g) eingenommen hatte [60].

## Verbraucherschutzmaßnahmen

### Höchstmengen für Vitamine und Mineralstoffe

In Artikel 5 der Richtlinie 2002/46/EG [11] und in Artikel 6 der VO (EG) Nr. 1925/2006 wird die KOM ermächtigt, für Vitamine und Mineralstoffe, die NEM oder anderen Lebensmitteln zugesetzt werden, Höchstmengen festzulegen und dabei Folgendes zu berücksichtigen:

1. *die sicheren Höchstgehalte *(also: ULs), die durch eine *wissenschaftliche Risikobewertung* auf der Grundlage allgemein anerkannter wissenschaftlicher Daten festgelegt werden, wobei die *unterschiedliche Empfindlichkeit der einzelnen Verbrauchergruppen* zu berücksichtigen ist;
2. die *Aufnahmemengen *an Vitaminen und Mineralstoffen, die *aus anderen Ernährungsquellen* zugeführt werden;
3. die *Bevölkerungsreferenzzufuhr* (also: Zufuhrreferenzwerte) für Vitamine und Mineralstoffe.

Folglich sollte die Höchstmengenfestsetzung so erfolgen, dass

- die Nährstoff-Gesamtzufuhr den jeweiligen UL nicht überschreitet und
- eine ausreichende Nährstoffzufuhr für alle Bevölkerungsgruppen sichergestellt ist.

Daraus ergibt sich, dass für jeden Nährstoff ein „sicherer Zufuhrbereich“ zu ermitteln ist, der nach unten durch den jeweiligen Zufuhrreferenzwert und nach oben durch den UL begrenzt wird.^6^

#### Aktuelle Entwicklungen auf EU-Ebene.

Nachdem die KOM im Jahr 2006 Arbeiten an Höchstmengen für Vitamine und Mineralstoffe auf EU-Ebene eingeleitet hatte, die schon 2009 aus verschiedenen Gründen wieder auf Eis gelegt wurden, hat die Arbeitsgruppe für Nahrungsergänzungsmittel und angereicherte Lebensmittel der KOM (KOM-AG^7^) auf Initiative Deutschlands im Jahr 2020 eine Ad-hoc-Taskforce einberufen, die nun wieder an dem Thema arbeitet. Die Taskforce ist eine von der KOM-AG eingerichtete Untergruppe von Experten, die von den EU-Mitgliedsstaaten benannt wurden und unter dem Vorsitz der KOM die wissenschaftlichen Aspekte, die bei der Höchstmengenableitung zu berücksichtigen sind, prüfen. In der Taskforce sind Experten aus Belgien, Dänemark, Deutschland, Frankreich, Griechenland, Irland, den Niederlanden, Norwegen, Polen, Spanien und der Tschechischen Republik vertreten.

Zunächst hat die Taskforce Vitamine und Mineralstoffe identifiziert, die von der EFSA bis 2024 neubewertet wurden; dies betraf Vitamin B6, Folsäure/Folat, Vitamin D, Mangan, Eisen, Vitamin A, β‑Carotin und Vitamin E [62]. Weiterhin wurde unter Berücksichtigung vorhandener Modelle ein Verfahren zur Ableitung von Höchstmengen erarbeitet, das die o. g. Prinzipien berücksichtigt. Die für NEM *und* angereicherte Lebensmittel zur Verfügung stehende „sichere Zufuhrmenge“ wird dabei berechnet als Differenz zwischen dem UL und der Nährstoffzufuhr im 95. Perzentil über die übliche Ernährung (ohne NEM und Anreicherung). Auf diese Weise können auch Menschen mit einer hohen Basis-Nährstoffzufuhr vor einer überhöhten Zufuhr geschützt werden (siehe auch [36]).

Die Beratungen auf EU-Ebene sind noch nicht abgeschlossen; wann also mit der Festsetzung von Höchstmengen gerechnet werden kann, ist zurzeit nicht absehbar. Solange keine EU-harmonisierten Höchstmengen existieren, können die EU-Mitgliedsstaaten nationale Regelungen anwenden. Dies wurde von einigen Mitgliedsstaaten bereits umgesetzt. Das BfR hat mit seinen Höchstmengenempfehlungen [33] zur Diskussion auf EU-Ebene beigetragen und zugleich eine Basis dafür geschaffen, auch in Deutschland ggf. nationale Höchstmengen festzusetzen.

### Einstufung „sonstiger Stoffe“ mit ernährungsspezifischer oder physiologischer Wirkung

Um die Aufnahme von „sonstigen Stoffen“ nach dem Verfahren gemäß Artikel 8 der VO (EG) Nr. 1925/2006 zu beschleunigen, wurde 2020 die *„Heads of Agencies Working Group on Food Supplements“* (HoA WG FS) unter dem Vorsitz des Bundesamts für Verbraucherschutz und Lebensmittelsicherheit (BVL) und Irlands (bis 2022) bzw. den Niederlanden (seit 2022) gegründet, in der Vertreter aus über 25 EU-Mitgliedsstaaten und des Europäischen Wirtschaftsraumes (EWR) mitarbeiten. Die HoA WG FS hat 2024 einen Bericht mit einer Liste von 117 Stoffen veröffentlicht, die aufgrund ihres Risikos für die menschliche Gesundheit nicht oder nur mit Beschränkungen in Lebensmitteln verwendet werden sollten [63].

Dreizehn dieser Stoffe wurden als prioritär eingestuft, weil davon auszugehen ist, dass sie Gesundheitsrisiken bergen, insbesondere wenn ihre Aufnahme über NEM weit über den durch die übliche Ernährung erzielten Mengen liegt: Cumarin in pflanzlichen Zubereitungen, Curcumin in Curcuma-Zubereitungen, *Hypericum perforatum*, ätherische Öle aus Melaleuca, Melatonin, Piperin, P‑Synephrin in Zitruszubereitungen, Tryptophan, *Actaea racemosa, Lepidium meyenii, Ocimum tenuiflorum, Tribulus terrestris, Withania somnifera*. Diese 13 Stoffe wurden der KOM zur Einleitung des Artikel-8-Verfahrens übermittelt [63]. Darüber hinaus wurden 65 der 117 Substanzen von der Arbeitsgruppe vorläufig als „neuartig“ und 49 als „nicht neuartig“ bzw. als „nicht neuartig in NEM“ eingestuft [63].

Einen ähnlichen Ansatz wie die HoA WG FS verfolgt auf nationaler Ebene die „*Arbeitsgemeinschaft Stoffliste*“, die sich aus Behördenvertretern und externen Experten aus Deutschland, Österreich und der Schweiz zusammensetzt. Die Gruppe erarbeitet unter dem Vorsitz des BVL eigene Stofflisten als Orientierungshilfe für die Lebensmittelüberwachung und -unternehmer [64]. Der Schwerpunkt liegt auf der Einstufung von einzelnen Stoffen auf Basis bekannten Wissens zu den Stoffen hinsichtlich ihrer Verwendung als Arzneistoff oder Lebensmittel sowie ihrer Neuartigkeit. Die Einstufung erfolgt mithilfe eines Entscheidungsbaums, der eine konsistente und eindeutige Zuordnung unter Berücksichtigung der aktuellen Rechtslage erlaubt. Bislang wurde von der Arbeitsgemeinschaft (AG) eine Pflanzenliste mit ca. 900 Einträgen, eine Pilzliste mit ca. 300 Einträgen und eine Algenliste mit ca. 300 Einträgen erarbeitet [64]. Die Listen liefern einen Überblick über kritische Inhaltsstoffe und die damit verbundenen Risiken; es ist jedoch nicht Ziel der AG, umfassende Risikobewertungen zu einzelnen Stoffen zu erarbeiten. Auch sind die Listen weder rechtsverbindlich noch abschließend; eine Abweichung von der vorgenommenen Einstufung muss aber gegenüber der zuständigen Lebensmittelüberwachungsbehörde begründet werden.

## Fazit

Eine Supplementierung von Vitaminen und/oder Mineralstoffen ist bei der gesunden Bevölkerung im Allgemeinen nicht nötig, erhöht aber, vor allem bei gut versorgten Personen, das Risiko für eine übermäßige Zufuhr. Nur unter bestimmten Bedingungen und für bestimmte Personengruppen wird eine gezielte Nahrungsergänzung empfohlen. Prinzipiell sollten Lebensmittel, insbesondere Nahrungsergänzungsmittel, denen Mikronähr- und/oder „sonstige Stoffe“ zugesetzt sind, nicht unkritisch verwendet werden. Die bestehenden Regelungslücken stellen sowohl Behörden, Fachkreise und Verbraucher, aber auch die Lebensmittelunternehmer vor Herausforderungen. Um Verbraucher bestmöglich zu schützen, sind harmonisierte Höchstmengen und eine umfassende Regulierung von „sonstigen Stoffen“ auf EU- oder ggf. nationaler Ebene dringend erforderlich.

## Footnotes

1 KiESEL = Kinder-Ernährungsstudie zur Erfassung des Lebensmittelverzehrs.

2 EsKiMo = Ernährungsstudie als KiGGS-Modul.

3 KiGGS = Basisstudie zur Gesundheit von Kindern und Jugendlichen in Deutschland.

4 DEGS 1 = Welle 1 der Studie zur Gesundheit Erwachsener in Deutschland.

5 Der UL definiert die tägliche Zufuhrmenge eines Nährstoffs aus allen Quellen, die bei chronischer Zufuhr mit hoher Wahrscheinlichkeit nicht mit unerwünschten gesundheitlichen Wirkungen einhergeht.

6 Also: Gesamtnährstoffzufuhr = Zufuhr aus üblicher Ernährung einschließlich NEM und/oder angereicherte Lebensmittel ≥ Zufuhrreferenzwert und Gesamtnährstoffzufuhr ≤ UL.

7 Arbeitsgruppe aus Vertretern von EU-Mitgliedsstaaten (engl. Working group on food supplements and on the addition of vitamins and minerals and of certain other substances to foods), die die KOM zu Rechtsetzungsvorhaben beraten soll.
